# Do Not Huff, Puff, or Vape That Stuff: Interstitial Airspace Disease in a Teenager

**DOI:** 10.1155/2020/8822362

**Published:** 2020-12-02

**Authors:** Amee A. Amin, Erica Haught, Youmna Mousattat

**Affiliations:** ^1^Charleston Area Medical Center Women & Children's Hospital, Charleston, WV, USA; ^2^WVU Department of Pediatrics, Morgantown, WV, USA; ^3^West Virginia University School of Medicine, Morgantown, WV, USA

## Abstract

A 17-year-old previously healthy male was admitted to the hospital for intractable and persistent vomiting, fever, cough, abdominal pain, and intermittent diarrhea and dehydration. He presented with severe chest pain and O_2_ desaturations up to 80% on room air. An infectious (including a nasopharyngeal swab), GI, and cardiac workup was completed and was negative except for elevated inflammatory markers with a C-reactive protein (CRP) level of 261 mg/L, erythrocyte sedimentation rate (ESR) of 53 mm/hr, and a D-dimer level of 0.93 mcg/ml. Chest X-ray showed diffuse multifocal infiltrates. The patient was treated with ceftriaxone and azithromycin initially for a suspected pneumonia. He was also started on 4L of nasal cannula O_2_ supplementation. Due to persistent hypoxic respiratory failure, worsening respiratory distress clinically, with tachypnea and retractions, and lab findings of elevated D-dimer, a chest CT was performed to rule out a pulmonary embolism (PE). Computed tomography (CT) findings were negative for PE but notable for diffuse airspace opacities, primarily within the lower lobes, with a ground-glass appearance concerning for ARDS. Upon further investigation of the social history, the patient admitted to vaping nicotine products for the past 4 years and tetrahydrocannabinol (THC) products within the last several months. He was immediately started on prednisone 30 mg BID for a diagnosis of e-cigarette or vaping product use-associated lung injury (EVALI) and started showing clinical improvement. The patient was able to be weaned off of supplemental oxygen to room air, and clinical symptoms of respiratory distress began to improve over the next 24 hours.

## 1. Introduction

Vaping is the process of inhaling an aerosol that is created by heating a liquid or wax-containing substance such as nicotine, cannabinoids, flavoring, and additives. A vaporizer is a battery-powered device with a heating element that produces this aerosol or vapor that users inhale [1]. The liquid that is heated can have a variety of flavors with or without the addition of toxic substances such as nicotine and cannabinoids. Over 7,700 flavors are available currently, marketed towards the youth especially [2]. E-cigarettes and vaporizers were initially introduced in the market to encourage quitting of cigarette smoking. However, with the variety of flavorings and the electronic nature of the cigarettes/vaporizers, adolescents have been attracted and form a large part of the consumer pool. Recent studies have also shown that the use of e-cigarettes furthers progression to use of regular cigarette smoking as well [3]. In this case, we present a unique case presentation of vaping-associated lung injury and treatment approaches.

## 2. Case Report

A 17-year-old male presented to the emergency department (ED) with fever, cough, congestion, nausea, and vomiting. He was seen by his pediatrician 3 days prior and was given azithromycin and oseltamivir albeit a negative rapid influenza test as well as a negative *Streptococcus pyogenes* test. His vomiting and nausea had worsened since starting oseltamivir with almost no per oral tolerance, and hence he was brought to the ED for fluid resuscitation and IV nausea medication. The patient also mentioned experiencing intermittent diarrhea for months, especially associated with food intake. He denied any chest pain, shortness of breath, palpitations, dizziness, lightheadedness, weakness, numbness, tingling, constipation, dysuria, hematuria, hematochezia, and melena. Physical examination revealed mild-to-moderate dehydration with dry oral mucosa. At the ED, patient's oseltamivir was discontinued, and his fluids were replenished. His leukocyte count was 12,000 cells/mm^3^, erythrocyte sedimentation rate (ESR) was 53 mm/hr, and chest X-ray showed mild infiltrates versus artifact. He was sent home on ondansetron and instructed to continue the azithromycin and follow-up with his pediatrician within 1 week and return to the ED for worsening symptoms.

**Figure 1:**
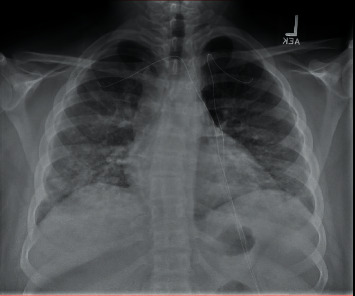
Chest X-ray showing interstitial opacities throughout the perihilar and basal lung regions.

The patient returned to the ED 2 days later with profuse vomiting, diarrhea, and abdominal pain worse in the right lower quadrant. Inflammatory markers at this time included a C-reactive protein (CRP): 260 and an ESR: 79. He was given fluids, ondansetron and morphine, to treat his nausea and pain, respectively. A computed tomography (CT) with intravenous contrast of his abdomen and pelvis showed no acute intra-abdominal findings but a possible pneumonitis in the lung bases bilaterally. Upon returning from the CT, his oxygen saturation was 80% on room air. He was placed on 3L nasal cannula with immediate improvement to 95% saturation. At this visit, the patient finally admitted to cough and intermittent shortness of breath. A chest X-ray (Figure 1) at this time showed multifocal pneumonia. He was started on ceftriaxone and azithromycin and admitted to the pediatric service.

**Figure 2:**
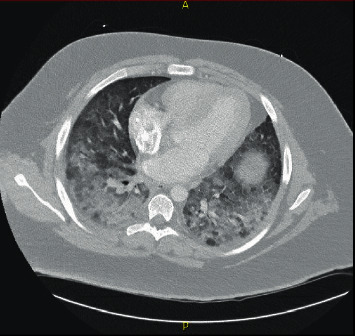
Chest CT angiography showing diffuse symmetric ground-glass opacities with confluent consolidation within both lower lobes.

Upon admission to the pediatric service, the patient endorsed fevers, sore throat, shortness of breath, right lower and substernal chest pain, nausea, vomiting, and diarrhea and denied any recent travel, exposure to sick contacts, anxiety, depression, rashes, hematuria, or hematochezia. A comprehensive cardiac, gastrointestinal, and infectious workup was completed including stat labs for complete blood count (CBC), basic metabolic panel (BMP), blood culture, *Mycoplasma pneumoniae* titers, urinalysis, stat echocardiogram (ECHO), electrocardiogram (EKG), troponins and B-type ventricular natriuretic peptide (BNP) with cardiac consult, D-dimer, and gastrointestinal stool panel. At this time, the patient continued to remain on 3L supplemental oxygen to maintain saturations above 90%. Laboratory results revealed a normal white blood cell count (WBC), negative blood cultures and mycoplasma titers, hypokalemia, dehydration, acute kidney injury (AKI), unremarkable EKG, ECHO, troponins and BNP, and an elevated D-dimer at 0.93 mcg/ml. Overnight, the patient was continued on appropriate IV fluids and oral potassium chloride (KCl) to correct his dehydration status, AKI, and hypokalemia. The patient continued to exhibit worsening respiratory distress clinically with oxygen support titrated up to 6L. A chest CT angiography (Figure 2) was performed which ruled out a pulmonary embolus but was notable for diffuse airspace opacities, primarily within the lower lobes, with a ground-glass appearance concerning for acute respiratory distress syndrome. At this time, the patient had been weaned down to 4L supplemental oxygen. Though the nausea and vomiting were controlled with IV ondansetron, diarrheal episodes persisted. GI panel at this time was negative.

Upon further investigation of his social history, the patient admitted to vaping nicotine products for the past 4 years and tetrahydrocannabinol (THC) products within the last several months. He was started on prednisone 30 mg twice a day and eventually weaned to 2L oxygen. The antibiotics were discontinued. Within 24 hours of initiating oral steroids, the patient was weaned to room air and was breathing well and comfortably, along with resolution of his diarrhea. Inflammatory markers also began trending down since admission.

The patient was diagnosed with e-cigarette or vaping product use-associated lung injury (EVALI). He was discharged on a steroid wean over 12 days and was agreeable to vaping cessation. The patient was followed outpatient with pulmonology 1 month after discharge with pulmonary function tests showing normal diffusion capacity and normal pre- and postbronchodilator spirometry, with no evidence of air trapping.

## 3. Discussion

Officially, the CDC recognized the entity of EVALI in August 2019, after physicians all over the country were beginning to identify severe to fatal lung infections in otherwise healthy individuals. A project called Monitoring the Future began in 2015 to survey the adolescent population in the USA, particularly middle and high school students in order to understand the prevalence of this new trending behavior and to identify the reasoning behind participating in the trend. Data from 382 public and private schools with a sample of 44,892 students identified the most important reasons for using an electronic vaporizer or e-cigarette. From “boredom,” to “quitting regular cigarettes,” to “wanting to experiment with a new trend,” or “even because it looks good,” the reasons were quite varied and socially influenced in this young age group. Earlier studies (2011–2014) were able to show a nine-fold increase among US high school students and a six-fold increase among middle school students in the use of e-cigarettes and vaporizers [2]. In a survey of 8th and 10th graders, there were at least twice as many students using electronic vaporizers (such as e-cigarettes), compared to regular cigarettes [4]. Curiosity, flavors, and peer influences were identified as top reasons for e-cigarette use in seven middle schools, high schools, and colleges in Connecticut [5].

In a study of college students in the New York State, enjoyment was listed as the primary reason for using e-cigarettes [6]. In a national study of teen flavored tobacco use, the primary reason for e-cigarette use was flavoring, followed closely by doing less harm than with cigarettes [7]. These studies suggest that reasons for vaporizer use among youth differ substantially from the reasons given by adults, who are more likely to report use of vaporizers in an attempt to quit smoking and improve personal health [8].

Though the specific cause of EVALI has not been determined, vitamin E acetate is a frequently seen ingredient used to thicken THC-containing products and has been detected in bronchoalveolar lavage samples in previously conducted studies in Minnesota [9, 10]. Other vape liquids also known as e-juice or e-liquid contain propylene glycol and glycerol as base ingredients which create the vapor. Thus, these additives and flavoring may be contributing to this significant lung injury, and some states have started to ban the use of these flavoring to help limit these issues. However, there is also concern that there is a negative impact of adolescent brain development affecting learning and attention [11]. This has now become a public health concern due to the potential negative impact on adolescent health. As a relatively recent clinical entity, there are no clear guidelines for management nor clear understanding of how and why the lung injury occurs. With such a varied clinical presentation nationwide, management has ranged from antibiotics for a community-acquired pneumonia like picture to steroid treatment for an anti-inflammatory acute respiratory distress syndrome (ARDS) like presentation. Most cases that reported using steroids have shown clinical improvement which supports the inflammatory ARDS picture, especially in previously healthy young adolescents who do not present with comorbidities that adults may have. Although our patient did not undergo flexible bronchoscopy with collection of bronchoalveolar lavage samples (BAL), it is an equally important diagnostic intervention which could assist in identifying other pulmonary interstitial pathologies. However, more research is still needed especially focusing on the evaluation and management of such cases. Accurate and timely diagnosis will go a long way towards reducing the use of unnecessary antibiotics as well as starting anti-inflammatory measures to reduce lung tissue damage. As far as general pediatrics is concerned, it is imperative that we are aware of the latest substance abuse devices and that we have a high index of suspicion especially while assessing adolescents and teens for such specific and new recreational substance delivery methods.

## Abbreviations

CRP: C-reactive protein
ESR: Erythrocyte sedimentation rate
CT: Computerized tomography
ARDS: Acute respiratory distress syndrome
THC: Tetrahydrocannabinol
EVALI: E-cigarette or vaping product use-associated lung injury.
